# Proteomic and network analysis characterize stage-specific metabolism in *Trypanosoma cruzi*

**DOI:** 10.1186/1752-0509-3-52

**Published:** 2009-05-16

**Authors:** Seth B Roberts, Jennifer L Robichaux, Arvind K Chavali, Patricio A Manque, Vladimir Lee, Ana M Lara, Jason A Papin, Gregory A Buck

**Affiliations:** 1 Center for the Study of Biological Complexity, Virginia Commonwealth University, Richmond, Virginia 23298, USA; 2 Department of Microbiology and Immunology, Virginia Commonwealth University, Richmond, Virginia 23298, USA; 3 Department of Biomedical Engineering, University of Virginia, Charlottesville, Virginia 22908, USA

## Abstract

**Background:**

*Trypanosoma cruzi *is a Kinetoplastid parasite of humans and is the cause of Chagas disease, a potentially lethal condition affecting the cardiovascular, gastrointestinal, and nervous systems of the human host. Constraint-based modeling has emerged in the last decade as a useful approach to integrating genomic and other high-throughput data sets with more traditional, experimental data acquired through decades of research and published in the literature.

**Results:**

We present a validated, constraint-based model of the core metabolism of *Trypanosoma cruzi *strain CL Brener. The model includes four compartments (extracellular space, cytosol, mitochondrion, glycosome), 51 transport reactions, and 93 metabolic reactions covering carbohydrate, amino acid, and energy metabolism. In addition, we make use of several replicate high-throughput proteomic data sets to specifically examine metabolism of the morphological form of *T. cruzi *in the insect gut (epimastigote stage).

**Conclusion:**

This work demonstrates the utility of constraint-based models for integrating various sources of data (e.g., genomics, primary biochemical literature, proteomics) to generate testable hypotheses. This model represents an approach for the systematic study of *T. cruzi *metabolism under a wide range of conditions and perturbations, and should eventually aid in the identification of urgently needed novel chemotherapeutic targets.

## Background

The increasing availability of complete genome sequences has spurred efforts to model biological systems on a comprehensive scale [1,2]. Constraint-based modeling has emerged in the last decade as a useful approach to the integration of genomic and other high-throughput data sets with more traditional, experimental data acquired through decades of biochemical and molecular research [3,4]. To date, constraint-based modeling has been extensively applied to probe the function of intracellular metabolism, although the constraint-based framework is in principle applicable to any set of chemical transformations, including signal transduction networks [5] and transcription or translation [6]. When combined with a specific method of analysis, e.g., flux balance analysis (FBA), constraint-based models can be used to generate quantitative predictions (e.g., growth rate of an organism) and yield testable hypotheses for future experimental investigations [7]. This permits an iterative process of model development, hypothesis generation and testing, and further model development and refinement [8]. The principles and methods of building and analyzing constraint-based models have been comprehensively reviewed in the literature [6,9,10]. Experimentally validated constraint-based models are providing integrative, systems-level views of the functioning of different metabolic networks of various organisms across a wide range of specific conditions (e.g., gene deletions, pharmacological interventions and environmental perturbations) [11-14].

*Trypanosoma cruzi *is a protozoan parasite of the order Kinetoplastida that infects humans and a wide variety of other mammals. Like other members of its order, *T. cruzi *is characterized by a single mitochondrion containing a complex network of DNA fibrils known as the kinetoplast [15]. *T. cruzi *displays many unusual biological features: specialized intracellular compartments, such as the glycosome (in which the initial reactions of glycolysis occur) [16,17], acidocalcisome [18], and reservesome [19]; widespread RNA editing of mitochondrial transcripts [20]; polycistronic transcription [21]; and trans-splicing [22], to name a few. Its life cycle is complex, involving multiple distinct morphologic stages in both its mammalian hosts and the triatomine insect vectors [15]. *T. cruzi *is the causative agent of Chagas disease, a potentially lethal condition affecting the cardiovascular, gastrointestinal, and nervous systems of the human host. The impact of Chagas disease is significant; approximately 10 million persons are affected, primarily in Latin America [23], and life expectancy is reduced by 9 years in those patients who develop chronic symptoms [24]. Despite decades of research, only two drugs, nifurtimox and benznidazole, have proven useful in treating this disease [25]. However, the efficacy of these drugs for chronic Chagas disease is far below 100 percent [26,27] and both are associated with significant adverse effects, such as peripheral neuropathy and central nervous system toxicity [25].

To permit a systems-level understanding of this parasite and to provide a basis for detailed modeling of host-parasite interactions, we present a validated, constraint-based model of *T. cruzi *strain CL Brener core metabolism. The model, hereafter referred to as *i*SR215 (see Methods for model naming convention), includes four compartments, 51 transport reactions, and 93 metabolic reactions covering carbohydrate, amino acid, and energy metabolism. In addition, we make use of several replicate high-throughput proteomic data sets to specifically examine metabolism of the morphological form of the parasite in the insect gut, or epimastigote stage of *T. cruzi *(see Methods for details on *T. cruzi *life-cycle). In doing so, we demonstrate the utility of constraint-based models for integrating various sources of information (e.g., genomics, primary biochemical literature, proteomics) to generate testable hypotheses. Previous work has used sequence analysis of the *T. cruzi *genome to produce improved annotations and thus extend our understanding of *T. cruzi *metabolism [28,29]. However the model reported here is the first constraint-based model of *T. cruzi *of which we are aware. It represents an approach to the systematic study of *T. cruzi *metabolism under a wide range of conditions and perturbations for the identification of novel chemotherapeutic targets.

## Results

### Properties of *i*SR215

The *i*SR215 network reconstruction accounts for the function of 215 genes and includes 162 reactions, of which 144 are metabolic reactions and 18 are exchange reactions (Table 1, see also additional file 1: DetailedResults.xls, for model in spreadsheet form and additional file 2: tcr.xml, for model in SBML format). Of the exchange reactions, 17 are input-output exchanges that allow metabolites to enter and/or leave the model system, and one is the biomass demand reaction used to drain metabolites assumed critical to the growth of *T. cruzi *(e.g., glucose-6-phosphate, pyruvate and oxaloacetate). Of the reactions in *i*SR215, 76 are supported by both genomic and direct biochemical evidence (e.g., enzyme activity measured) in *T. cruzi*, and 100 reactions are supported by genomic or literature-derived evidence in *T. cruzi *or related organisms. Most reactions not associated with literature-based evidence are intracellular transports, reflecting the fact that little is known about such processes in *T. cruzi*.

**Table 1:** Properties of *i*SR215

Property	Count
Genes	215
Reactions	162
Gene associated	92
Non-gene associated (intracellular)	1
Non-gene associated (transport)	51
Exchange	18
Input-output	17
Demand (biomass)	1
Metabolites	158
Compartments	4
Literature References	182

**Figure 1:**
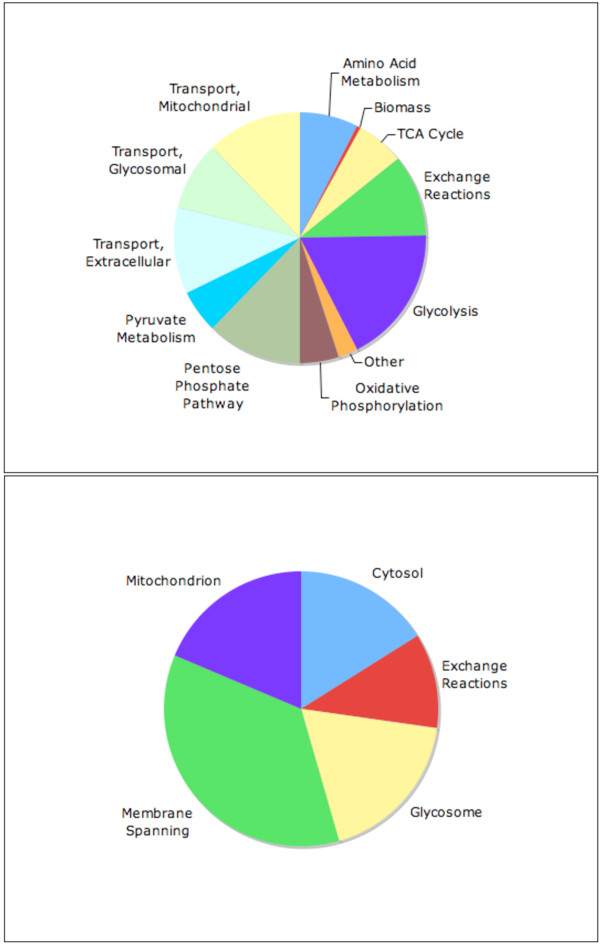
**Breakdown of *T. cruzi *core metabolic network**. Depiction of the content of iSR215 with reactions categorized by pathway (panel 1A) or by compartment (panel 1B). The model includes several of the core pathways of metabolism. Transport, or membrane spanning reactions are a sizeable fraction of all reactions since this is a multicompartment model of *T. cruzi *metabolism, including glycosomal and mitochondrial compartments.

Figure 1 provides an overview of the reactions of *i*SR215 as grouped by pathway and compartment. As illustrated in panel A, several central pathways of metabolism are fully or partially included: glycolysis, tricarboxylic acid (TCA) cycle, the pentose phosphate pathway, oxidative phosphorylation, and metabolism of various amino acids. Most reactions are part of carbohydrate metabolism (including glycolysis, the pentose phosphate pathway, and pyruvate metabolism). Further, as depicted in panel B, reactions are distributed across three intracellular compartments – cytosol, glycosome, and mitochondrion – and the extracellular compartment. The membrane-spanning group consists of reactions that involve the transfer of metabolites between subcellular compartments as well as between the cytosol and extracellular space. Because *i*SR215 contains four compartments, there are many membrane-spanning reactions included (a total of 58). The glycosome of *T. cruzi *is thought to be relatively impermeable to metabolites, especially to adenine nucleotides and NAD(H) [30]; thus, this compartment must be both energy and reduction-oxidation balanced. Accordingly, *i*SR215 contains no reactions transporting ATP/ADP/ATP or NAD(H) between the glycosome and cytosol, i.e., in any feasible solution, there is no net change in ATP/ADP ratio or in NAD+/NADH ratio within the glycosome. Furthermore, the model also accounts for substrate level and proton motive force coupled ATP synthesis, as well as the synthesis of various intermediate metabolites critical to the formation of biomass (e.g., acetyl-CoA).

### Defining epimastigote stage-specific metabolism

We used cultured *T. cruzi *epimastigotes to generate several large replicate proteomic data sets (see Methods). A total of 1047 distinct proteins were identified across 8 epimastigote samples. Many of the identified proteins were not associated with any well-defined function (660 proteins annotated as hypothetical). Of the identified proteins that were linked to functions, the most commonly occurring functional categories were metabolic processes (133) and translation (110). Some of the more commonly occurring functional subcategories of metabolic processes were: nucleobase, nucleoside, nucleotide and nucleic acid metabolic processes (68); amino acid and derivative metabolic processes (61); and carbohydrate metabolism (44). The complete proteomics results can be found in additional file 1: DetailedResults.xls.

The proteomics data were used to constrain *i*SR215 (the "full model," with all reactions available) to an epimastigote stage-specific model (the "epimastigote model," with some reaction fluxes set to zero). If there was no evidence for the expression of a given protein in the epimastigote stage, the upper and lower bounds of the corresponding reaction (catalyzed by the protein) were constrained to zero, thereby restricting the flux of the reaction to zero. In the case of a reaction catalyzed by an enzyme complex, we allowed the reaction to occur (i.e., did not constrain the flux to zero) if any protein component of the complex was detected by the proteomics experiment.

**Figure 2:**
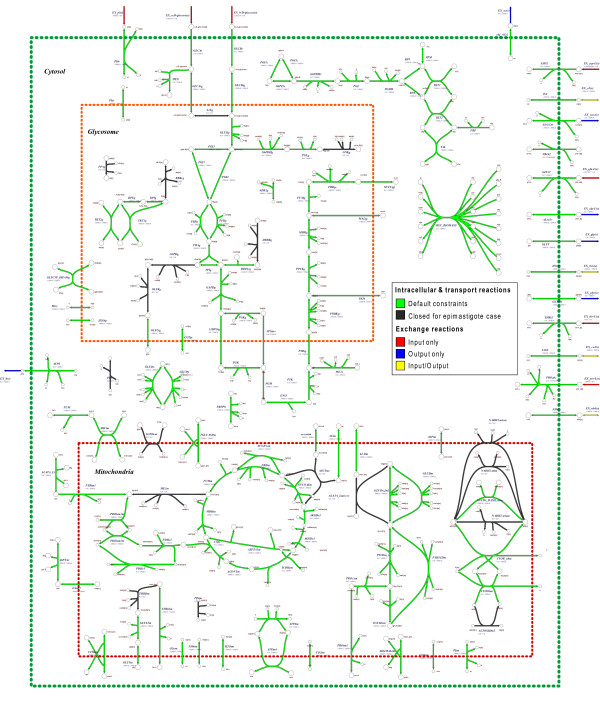
***T. cruzi *core metabolic network model, section**. A map illustrating a section of the core metabolic network model of *T. cruzi*. The model accounts for 144 intracellular and transport reactions, 17 input/output exchange reactions and 1 biomass demand reaction across four compartments: cytosol, mitochondria, glycosome and extracellular space. Reactions colored in green are present in both the full and epimastigote specific reconstructions. Those reactions present only in the full network and not present in the epimastigote (i.e. flux is constrained to zero) are depicted in black. Exchange reactions are shown in red (metabolites allowed to only enter the system), blue (metabolites allowed to only leave the system) and yellow (metabolites allowed to enter and/or leave the system). The map also indicates the lower and upper flux constraints for each reaction shown. For a depiction of the entire model, please see additional file 3: CruziCoreMetabolicNetwork.pdf.

A graphical depiction of a section of the compartmentalized reconstruction is shown in figure 2 (the entire reconstruction is depicted in additional file 3: CruziCoreMetabolicNetwork.pdf). All of the reactions illustrated in the map are present in the "full" model. However, the fluxes of the reactions colored in black are constrained to zero in the "epimastigote" case, effectively removing them from the network. Exchange reactions that allow metabolites to only enter the system from the surrounding environment are colored in red. Further, exchange reactions that allow metabolites to only leave the system are in blue, and those reactions that allow metabolites to either enter or leave the system are colored in yellow. Intracellular reactions that are colored green are associated with default constraints at the start of the simulation experiments.

Graphical representations of flux distributions calculated using the genome-based full model and the epimastigote, stage-specific model are depicted as additional files (additional file 4: FullModelFluxes.pdf and additional file 5: EpimastigoteFluxes.pdf). Comparison of these results reveals redistribution of fluxes involving multiple areas of the metabolic map. One example is substrate level phosphorylation in the mitochondrion. In the full model, ATP is generated by succinate-CoA ligase (SUCOASm) as part of a cycle involving acetate-succinate CoA-transferase (ASCTmr); in the epimastigote model, this same reaction generates ATP as part of the TCA cycle. This is due to the fact that ASCTmr flux is constrained to zero in the epimastigote model. Mitochondrial fumarate reductase (FRDm) activity increases from zero flux in the full model to relatively high flux in the epimastigote model. This is likely due to the inactivity of complex I in the epimastigote, with reoxidation of NADH largely taken over by FRDm. Correctly accounting for stage-specific features of metabolism is an important step to more accurately modeling metabolism.

### Reaction lethality predictions

*In silico *predictions of lethality can be made at the level of genes or reactions using constraint based models. Many of the reactions in *i*SR215 are associated with isozymes (see additional file 1: DetailedResults.xls). In these cases, *in silico *deletion of one gene will not result in a lethal prediction, even if the reaction itself is critical for the growth of the parasite. Thus, we focus here on the set of *reactions *predicted to be lethal, as this provides a better view of critical points in the network, i.e., those reactions which if blocked by a chemotherapeutic would lead to the inability of the parasite to replicate (a complete listing of reactions predicted to be essential can be found in the additional file 1: DetailedResults.xls).

All reactions in *i*SR215 were classified as lethal or nonlethal by systematically constraining the upper and lower flux bounds of each reaction to be zero flux, and attempting FBA under defined *in silico *medium conditions (see Methods). As shown in table 2, total of 26 reactions were predicted to be essential in the full model, and 40 were predicted to be essential in the epimastigote-specific model. Of the 14 reactions with different essentialities in the full and epimastigote models, three were the result of the zero flux constraint on aldose-1-epimerase in the epimastigote model. Without this capability, reactions that circumvent the inability to interconvert alpha- and beta-glucose anomers became critical. Four reactions had different essentialities that resulted from the zero flux constraint on glycosomal glycerol-3-phosphate dehydrogenase and glycerol kinase in the epimastigote model. Without these activities, reactions allowing the regeneration of NAD via glycosomal fumarate reductase became critical. Six reactions had different essentialities between the two networks because of the zero flux constraint on threonine dehydrogenase in the epimastigote model. In the absence of this activity, reactions permitting acetyl-CoA production via pyruvate became critical.

**Table 2:** Predicted reaction lethality

	Deletion Level	Lethal	Trival Lethal	NonTrivial Lethal	NonTrivial Total	Total Cases
Full Model	Single	26	0	26	145	145
	Double	1968	1872	96	8568	10440
Epimastigote	Single	40	0	40	145	145
	Double	3063	2880	183	7560	10440

We also simulated all possible double reaction deletions, i.e., simultaneous elimination of two reactions. In total, there were 10440 double deletions. In the full model, 1968 of these cases proved to be lethal. Most of these (1872) were "trivial" in that the double deletion involved at least one reaction that was lethal in a single deletion. There were 96 non-trivial double deletions, i.e., involving reactions that, while not lethal individually, were lethal when deleted together. In the epimastigote model, there were 3063 lethal double deletions, including 2880 trivial and 183 non-trivial cases. For a complete listing of all single and non-trivial double reaction deletions, see additional file 1: DetailedResults.xls. Each of these predictions represents a potentially testable hypothesis. Reactions that are experimentally shown to be lethal are of special interest, as these may correspond to novel chemotherapeutic targets.

### Validating network analysis with experimental data

As a simple check on the validity of *i*SR215 network reconstruction, we used the model to determine byproducts of metabolism under aerobic and anaerobic *in silico *culture conditions (see table 3). While there is some inconsistency in the literature, most experiments indicate that byproducts of *T. cruzi *metabolism include succinate [31-34], L-alanine [31,33], and CO_2 _[30]. Other byproducts that have been experimentally detected include acetate [31-33] and glycine [35]. These general observations are reproduced by *i*SR215. In the epimastigote model, CO_2_, succinate, and L-alanine are predicted byproducts of metabolism using defined *in silico *conditions. We also observed secretion of acetate and glycine using the full model. It is possible that different sets of byproducts could be secreted under alternative optimal solutions. To address this possibility, we used flux variability analysis [36]. In FVA, one seeks to find a range of flux values on specific reactions that are compatible with maximal growth. We found that the reactions draining succinate and L-alanine must have non-zero flux to achieve optimal growth in the epimastigote. In the full model, the flux on the reaction draining succinate must be non-zero. For the fluxes draining glycine and acetate, at least one of the two must have a non-zero value to achieve optimal growth.

**Table 3:** Predicted metabolic by products

Excreted	Full	Full anaerobic	Epimastigote	Epi anaerobic
acetate	X			
L-alanine			X	
co2	X		X	
glycine	X			
glycerol				
h+	X	X		X
h2o	X	X	X	X
nh4				
o2				
succinate	X	X	X	X

We next sought to validate *i*SR215 using available data on gene or gene product essentiality in *T. cruzi *or related species (see table 4) [37-51]. The experimental data were collected using a variety of techniques (e.g., RNAi based methods, pharmacological inhibition of the enzymatic action of a certain gene product). In all, 58 test cases were derived based on evidence from the literature, including both the full and epimastigote models. In 46 of these cases, our simulations correctly reproduced the experimental results, yielding 79.3% accuracy. All reactions that were nonlethal in published literature were correctly predicted as such in the case of the full model. There were three instances of reactions incorrectly predicted as nonessential in the full model (when compared to published data) that were corrected by the imposition of the epimastigote constraints: 1) glycosomal triose phosphate isomerase, 2) coinhibition of fumarate reductase and succinate dehydrogenase, and 3) coinhibition of pyruvate dehydrogenase and alpha-keto glutarate dehydrogenase. Three reactions were wrongly predicted to be essential in the case of the epimastigote model: 1) glycosomal fumarate reductase, 2) pyruvate dehydrogenase, and 3) pyruvate dehydrogenase and succinate dehydrogenase combined inhibition. Finally, there were three cases where our predictions were incorrect in both the full and epimastigote models: 1) malic enzyme, 2) pyruvate kinase, and 3) succinate – CoA ligase.

**Table 4:** Comparison of predicted and experimental reaction lethality.

Organism and stage	Experimental Method [ref]	Experimental Target	Model reaction(s) constrained	Finding from literature	Full model prediction	Epimastigote model prediction
bloodstream T. brucei***	drug-2-hydroxybenzaldehyde-5-phosphate [37]	fructose-1,6-bisphosphate aldolase	FBAg	lethal	lethal	lethal
T. cruzi amastigotes	drug- 6-phosphogluconate analogues [38]	phosphogluconate dehydrogenase	PGDH	lethal	lethal	lethal
T. cruzi amastigotes	drug- adenosine analogs [39]	glyceraldehyde-3-phosphate dehydrogenase	GAPDg	lethal	lethal	lethal
T. cruzi epimastigotes	drug- bisphosphonates [40]	hexokinase	HEXg, GLUKg	lethal	lethal	lethal
T. cruzi epimastigotes	monoclonal antibody [41]	triosephosphate isomerase	TPIg	lethal	nonlethal	lethal
T. cruzi epimastigotes	drug- peroxynitrite [42]	succinate dehydrogenase and fumarate reductase*	SUCD1rm**, FRDm, FRDgr	lethal	nonlethal	lethal
bloodstream T. brucei	RNAi [43]	phosphofructokinase	PFKg	lethal	lethal	lethal
bloodstream T. brucei	RNAi [43]	phosphoglycerate mutase	PGM	lethal	lethal	lethal
bloodstream T. brucei	RNAi [43]	enolase	ENO	lethal	lethal	lethal
procyclic T. brucei	RNAi [44]	malic enzyme	ME1x, ME1m	lethal	nonlethal	nonlethal
procyclic T. brucei	RNAi [45]	e1 alpha subunit of pyruvate dehydrogenase, 2-oxoglutarate dehydrogenase	PDHam1m, AKGDam1	lethal	nonlethal	lethal
bloodstream T. brucei	RNAi [43]	pyruvate kinase	PYK	lethal	nonlethal	nonlethal
procyclic T. brucei	RNAi [45]	succinyl-CoA synthetase (succinate CoA ligase)	SUCOASm	lethal	nonlethal	nonlethal
L. donovanni promastigotes	nontargeted gene disruption [46]	adenosine kinase	ADK1g	nonlethal	nonlethal	nonlethal
bloodstream T. brucei	RNAi [47]	alanine aminotransferase	ALATA_L, ALATA_Lm	nonlethal	nonlethal	nonlethal
bloodstream T. brucei	RNAi [47]	cytochrome C oxidase subunit IV	CYOO6m	nonlethal	nonlethal	nonlethal
bloodstream T. brucei	RNAi [47]	glycerol-3-phosphate dehydrogenase (FAD)	G3PDcm	nonlethal	nonlethal	nonlethal
procyclic T. brucei	RNAi [45]	e1 subunit of 2-oxoglutarate dehydrogenase	AKGDe1	nonlethal	nonlethal	nonlethal
procyclic T. brucei	RNAi [44]	F1 complex of ATP synthase	ATPSm	nonlethal	nonlethal	nonlethal
procyclic T. brucei	RNAi [48]	glycosomal NADH-dependent fumarate reductase	FRDgr	nonlethal	nonlethal	lethal
procyclic T. brucei	RNAi [44]	mitochondrial fumarate reductase	FRDm	nonlethal	nonlethal	nonlethal
procyclic T. brucei	RNAi [45]	e1 alpha subunit of pyruvate dehydrogenase	PDHam1m	nonlethal	nonlethal	lethal
procyclic T. brucei	RNAi [45]	e1 alpha subunit of pyruvate dehydrogenase, succinate dehydrogenase	PDHam1m, SUCD1rm	nonlethal	nonlethal	lethal
procyclic T. brucei	RNAi [44]	phosphoenolpyruvate carboxykinase	PPCKg	nonlethal	nonlethal	nonlethal
procyclic T. brucei	RNAi [44]	proline oxidase	PRO1xm	nonlethal	nonlethal	nonlethal
procyclic T. brucei	RNAi [45]	succinate dehydrogenase	SUCD1rm	nonlethal	nonlethal	nonlethal
bloodstream T. brucei	targeted gene disruption [49]	aconitase	ACONTm	nonlethal	nonlethal	nonlethal
procyclic T. brucei	targeted gene disruption [50]	acetyl:succinate CoA-transferase	ASCTmr	nonlethal	nonlethal	nonlethal
procyclic T. brucei	targeted gene disruption [51]	pyruvate phosphate dikinase	PPDKg	nonlethal	nonlethal	nonlethal

*Hypothesized to be the main targets of peroxynitrite in *T. cruzi*

**Succinate dehydrogenase is represented in the model by two reactions, SUCD1rm, and SUCD3-u6m; inhibition of succinate dehydrogenase simulated by inhibiting the first of these two

***One variation of compound was lethal for *T. cruzi*, as well

See references [37-51] for details.

## Discussion

Our first attempts at integrating the metabolic network reconstruction with epimastigote stage proteomic data provide a specific example of how constraint-based models can serve to help refine/interpret existing data. Initially, when the additional constraints arising from the proteomic data were imposed on the genome-based reconstruction, no solution was possible by FBA. By mapping these constraints on to *i*SR215, we determined that they resulted in the loss of three critical enzyme activities: fumarate hydratase, ribulose-5-phosphate 3-epimerase, and glucose-6-phosphate 1-dehydrogenase. The latter two reactions are part of the pentose phosphate pathway, leading directly to the production of key components for the biomass demand reaction. Subsequently, we found biochemical evidence for the activity of these enzymes in epimastigotes [52]. Thus, we removed the imposed constraints on ribulose-5-phosphate 3-epimerase and glucose-6-phosphate 1-dehydrogenase. Fumarate hydratase was the only enzyme of the TCA cycle not detected in the proteomics experiments. Since the TCA cycle is known to be active in *T. cruzi *epimastigotes [53], we decided it was reasonable to remove the imposed zero flux constraint from this reaction, as well. By making these three adjustments, we obtained a positive growth rate by FBA. Thus, the constraint-based model helped identify three cases of probable false negative findings from the proteomics experiments. By providing a framework for the systematic consideration of the functional relationships between components, the model allowed us to make more informed judgments on the validity of the proteomic data. An algorithmic method for integrating gene expression data into analysis of constraint-based models was recently published [54]. Approaches such as this will likely be invaluable for integrating proteomic and transcriptomic data with future iterations of *i*SR215, especially as the scope of the model increases.

Our attempts to validate the model by comparing predicted and experimentally observed reaction lethality should all be interpreted in light of three caveats. First, as noted in Table 4, most of these comparisons are made, not to *T. cruzi*, but to related trypanosomatid species. All species used for our comparisons are metabolically quite similar [30], but there are differences [32]. Second, there are certain limitations that arise from the fact that *i*SR215 is not a genome-scale model; these are discussed in more detail below. Third, the experimental data used were collected using complex, undefined culture media that we could not reproduce *in silico*. The overall accuracy of our predictions (79.3%) is comparable to that reported for other organisms, e.g., 83% for *Saccharomyces cerevesiae *[4], 70% for *Leishmania major *[55], 91% for *Escherischia coli *[56].

We found three instances of reactions incorrectly predicted as nonessential in the full model that were corrected by the imposition of the epimastigote constraints when compared with published literature (see Table 4): 1) glycosomal triose phosphate isomerase, 2) coinhibition of fumarate reductase and succinate dehydrogenase, and 3) coinhibition of pyruvate dehydrogenase and alpha-keto glutarate dehydrogenase. This observation suggests that the additional epimastigote constraints correctly eliminate certain steady state metabolisms that would otherwise be available to the parasite according to the full model. It is noteworthy that cases 1) and 2) are the only ones in which available experimental data were collected specifically in *T. cruzi *epimastigotes, as opposed to other morphological stages or related trypanosomatid species.

Three activities were wrongly predicted to be essential in the case of the epimastigote model when compared to published data (see Table 4): 1) glycosomal fumarate reductase, 2) pyruvate dehydrogenase, and 3) pyruvate dehydrogenase and succinate dehydrogenase combined inhibition. Of course, if pyruvate dehydrogenase is predicted to be essential, it is to be expected that inhibition of additional reactions (e.g., succinate dehydrogenase) along with pyruvate dehydrogenase would also be predicted as essential. We left these as independent predictions since we did not know *a priori *whether neither, one, or both would be predicted as essential. Thus 3) is incorrect because 2) is incorrect. The reason that 2) is incorrect is that without pyruvate dehyrogenase, there is no way to produce acetyl-CoA in the core metabolic model (and no other route for consuming CoA produced in the mitochondrion). Adding an additional source of acetyl-CoA and sink for mitochondrial CoA results in a positive-growth solution by FBA. Threonine dehydrogenase, which might otherwise produce acetyl-CoA is inactivated in the epimastigote model. Glycosomal fumarate reductase is also incorrectly predicted to be essential in the epimastigote case. The reason for this incorrect prediction is that without glycosomal fumarate reductase, there is no existing route to stoichiometrically convert NADH back to NAD inside the glycosome. Adding such a process back to the model results in a positive-growth solution by FBA. Glycerol-3-phosphate dehydrogenase and glycerol kinase could fill this role, but they are inactivated in the epimastigote model. Thus, these incorrect predictions may be either due to the incomplete nature of the model (e.g., another route for acetyl-CoA production that is not represented in *i*SR215) or perhaps because of possible changes in protein expression that are not explicitly represented in the model (e.g., glycerol-3-phosphate dehydrogenase and glycerol kinase expression may be conditionally induced in epimastigotes).

Finally, there were three examples in which our predictions were incorrect in both the full and epimastigote models: 1) malic enzyme, 2) pyruvate kinase, and 3) succinate – CoA ligase. Here again, these discrepancies are likely due to the fact that the core model remains incomplete. Specifically, we have not attempted to fully account for proton gradients in *T. cruzi*. Therefore, synthesis via oxidative phosphorylation is coupled not only to the pumping of hydrogen ions by the respiratory chain but also to the production of hydrogen ions by cytoplasmic processes. Thus, the contribution of oxidative phosphorylation to total ATP synthesis suggested by *i*SR215 is likely to be an overestimate of the true contribution. In fact, the majority of ATP synthesis in these parasites is thought to be via substrate level phosphorylation [30]. This explanation seems to account for the incorrect prediction in the case of succinate – CoA ligase; when mitochondrial ATP synthase is constrained to zero flux, the succinate – CoA ligase reaction is essential. The reasons for the incorrect prediction for malic enzyme and pyruvate kinase are less clear. Again, it may be a reflection of the incomplete nature of the core model, i.e., there are processes not included in *i*SR215, which are dependent on the production or consumption of metabolites that these reactions entail. Overall, the accuracy of the model at this stage is encouraging.

## Conclusion

We have presented the first constraint-based metabolic model and analysis of *T. cruzi*, the protozoan parasite responsible for Chagas disease. This model accounts for central metabolic processes such as ATP generation and production of key intermediate metabolites. Moreover, the model includes three major intracellular compartments: the glycosome (a characteristic feature of *T. cruzi *and other trypanosomes), the mitochondrion, and the cytosol. Thus, many key features of trypanosomatid metabolism are represented. Most reactions in the model are supported by both direct biochemical evidence from the primary literature and by the genome annotation of *T. cruzi*. Many aspects of the cellular physiology and data concerning gene/reaction essentiality are accurately captured by *i*SR215. Future work on *i*SR215 will initially be focused on expanding the scope of the model to include all known *T. cruzi *metabolic reactions using procedures similar to those described here. Previous experience suggests that as the model is expanded and refined through successive iterations, the accuracy of the essentiality predictions will improve [3,57-59]. We have used a wide variety of evidence in constructing this model, namely genomic, proteomic, and information drawn from primary literature. This gives us a fairly high level of confidence in what is currently included in the model.

Our incorporation of several replicate high-throughput proteomic data sets illustrates the integrative capabilities of constraint-based models. This data was used to further constrain the space of possible solutions and enabled us to examine stage-specific aspects of metabolism. In the available instances where experimental confirmation was available for *T. cruzi *epimastigotes specifically, applying the proteomic constraints produced the correct prediction; without these additional constraints, the model prediction was incorrect. Constraints reflecting data on gene expression have previously been shown to improve the accuracy of constraint-based model predictions [60,61]. Our experience also indicates that constraint-based models may aid in the interpretation of such high-throughput datasets, potentially helping to identify false negative results. Proteomics data are imperfect, and false negative results tend to be more of a concern than false positives. We attempted to minimize false negatives experimentally by using sub-cellular proteomics techniques (see Methods). This approach proved quite useful, as the number of identified proteins increased by more than 50% over traditional shotgun-based methods (data not shown). Even so, there were three examples of probable false-negative findings that were revealed by considering the proteomics data in light of *i*SR215 (see discussion of fumarate hydratase, ribulose-5-phosphate 3-epimerase, and glucose-6-phosphate 1-dehydrogenase, above). Thus, constraint-based models can provide a systematic framework for the interpretation and evaluation of findings from proteomics experiments.

Our comprehensive gene and reaction deletion testing illustrates the way in which constraint based models can become tools for drug discovery. Each of these predictions represents a testable hypothesis. Assuming that at least some of the reactions predicted to be essential are experimentally verified, these suggest points of vulnerability in the parasite's metabolism, which could potentially be exploited to develop new, urgently needed chemotherapeutics. Additionally, model-based predictions of the effect of combined environmental manipulations and specific reaction inhibitors can be generated using this model.

## Methods

### Metabolic Reconstruction

The theoretical basis and procedures underlying construction and analysis of constraint-based models have been extensively reviewed [6,8]. The process of network reconstruction involves data collection, metabolic reaction list generation, and determination of gene-protein-reaction relations. The sources of information used for our network reconstruction were: publicly available annotations of the *T. cruzi *genome (UniProt [62], KEGG [63], GeneDB [64]), our in-house annotation of the *T. cruzi *genome (JM Alves, unpublished), information from biochemistry textbooks and standard databases (e.g., KEGG), and primary biochemical literature on *T. cruzi *or closely related species (e.g., *Trypanosoma brucei*, *Leishmania major*). To identify relevant primary biochemical literature, we searched PUBMED for review articles on Trypanosomatid metabolism, with special focus on the pathways of central metabolism (e.g., search term "*Trypanosoma cruzi *glycolysis"). For individual reactions under consideration, we searched using a common variant of the reaction name, plus *T. cruzi *or the name of a related species (e.g., search term "*Trypanosoma cruzi *hexokinase"). We examined hundreds of articles in detail and screened the abstracts of many more. While we cannot claim to have exhausted the literature on *T. cruzi *metabolism, we searched to the point of reaching a high degree of confidence for most reactions in the model (i.e., that these reactions do, in fact, occur in *T. cruzi*). In some cases, references sought in connection with one reaction were found to report activity measurements corresponding to other model reactions. The recently published genome-scale constraint based model of *L. major *was also used as a guide in model construction and analysis [55]. These information sources were used to compile the metabolic reaction list, including reaction stoichiometry, reversibility, sub-cellular localization, and gene locus/loci for each reaction comprising core metabolism. The relations between genes, gene products and metabolic reactions were encapsulated in Boolean gene-product-reaction (GPR) statements (e.g., gene A codes for protein X or protein Y, protein X and protein P together catalyze reaction R, etc.).

We defined core metabolism as those metabolic reactions directly or closely related to energy generation (ATP) or involved in production of critical metabolites (see Biomass Equation, below). Transports between intracellular compartments were often represented as simple, bidirectional diffusion reactions, or occasionally as co-transports with hydrogen ions, as there currently are no data to suggest more specific mechanisms. This approach has proven useful as a first approximation for representing eukaryotic intracellular transport processes [4].

The glycosome is thought to be relatively impermeable to metabolites, especially to adenine nucleotides and NAD(H) [30]. Therefore in our reconstruction, we required that the glycosome be both energy balanced (ATP production balances ATP consumption) and reduction-oxidation balanced (NADH production balances NADH consumption). We imposed these constraints simply by excluding transports between the cytosol and glycosome for these and related (e.g., ADP, NAD+) metabolites.

Once the network was reconstructed, the list of metabolic reactions was transformed into a stoichiometric matrix (S), i.e., the mathematical representation of the reconstructed network. The dimensions of the S matrix are *m *× *n*, where *m *is the number of metabolites and *n *is the number of reactions. Each element S_ij _represents the stoichiometric coefficient of metabolite *i *in reaction *j*. If the metabolite in a particular reaction is a reactant, the coefficient is negative (the metabolite is consumed by the reaction); if it is a product, the coefficient is positive (the metabolite is produced by the reaction). The S matrix was then used as the basis of further analysis, i.e., Flux Balance Analysis (FBA, see below). Model construction and analysis were carried out using SimPheny (Genomatica, Inc.).

### Naming Convention

We follow the previously established convention of naming constraint-based models [3]. Model names begin with '*i*' to denote *in silico*, followed by the first author's first and last initials ('SR'), followed by the number of genes that are part of the model ('215').

### Flux Balance Analysis

Once constructed, the constraint-based model was analyzed using FBA. In essence, FBA uses linear programming (LP) based optimization to identify a particular flux distribution (a single point in the multidimensional space of possible metabolic behaviors) that optimizes a given metabolic objective (see Biomass Equation, below). The LP optimization problem is formulated as:

Z is the objective function; for this study, Z was defined as the biomass equation (see below). The second two statements are the flux constraints. **S **is the stoichiometric matrix defined above. Each reaction flux is subject to lower and upper bounds, as indicated (in cases where these are not known, a_i _and b_i _are set to some arbitrary numbers that exceed any feasible internal flux). The solution to this problem is an optimal flux distribution, **v**, a vector that contains flux values for each reaction in the network. The solution is optimal in the sense that it maximizes the flux through the objective Z.

Mathematically, **S **is a transformation of the reaction flux vector, **v**, to a vector of time derivatives for each metabolite concentration:

Since the time constants for metabolic transients are fast (< tens of seconds), but the time constants for cell growth are long (hours to days), the metabolites can be considered as existing in a quasi-steady state [6]. This leads to the second equation of the LP optimization problem above. Because of the emphasis on steady states, assumptions regarding reaction kinetics are not needed. Note also that *in silico *growth conditions can be defined via a_i _and b_i_. For example, if an analysis is to be conducted with glucose, but not glycerol, as an available metabolite, the flux for a process corresponding to glucose input would be assigned some nonzero positive maximal value, while the flux for a process corresponding to glycerol input would be constrained to be zero (a_i _= b_i _= 0). In all of our FBA simulations, we allowed the following extracellular metabolites to enter the system: glucose, glutamate, proline, aspartate, threonine, phosphate, CO_2_, water, and NH_4_; oxygen was allowed to enter in aerobic, but not anaerobic, simulations. The following extracellular metabolites were allowed to leave the system: succinate, alanine, glycine, acetate, glycerol, oxygen, water, CO_2_, NH_4_, and hydrogen.

We also incorporated information from the literature on maximal rate of uptake of glucose and amino acids where possible. For example, Vmax for glucose uptake *in T. cruzi *has been reported as ~46 nmol/min per mg of protein [65]. Given that ~47% of T. cruzi dry weight is protein [66], this corresponds to ~1.31 mmol/hr per gm dry weight. Since transport of both the alpha and beta anomers of glucose is explicitly represented in the model, we simply set the maximum flux (b_i_) on each reaction to ~0.65 mmol/hr per gm dry weight. In a similar fashion the maximum flux on glutamate uptake was set to ~0.027 [67], on proton symport-based proline transport to ~0.0036 [68], on ATP-dependant proline transport to ~0.020 [68], for aspartate uptake to ~0.0020 [69], and on threonine uptake to ~0.01 (approximation based on experimental values for the other transporters).

### Biomass Equation

In FBA, optimization is used to find a particular flux distribution that maximizes a given metabolic objective. Typical metabolic objectives chosen for optimization include maximization of ATP production, byproduct secretion, or biomass production. In our work, we chose to optimize for biomass production [70]. Biomass production is represented in our model by an additional metabolic reaction. Its reactants include such critical metabolites as ATP, acetyl-CoA, and NADPH; its products include ADP, inorganic phosphate, and other metabolites that represent byproducts of anabolic metabolism. This particular equation and its stoichiometric coefficients were derived from the biomass requirements for *Bacillus subtilis *[71]. These requirements were established for *B. subtilis *using detailed modeling and isotopic tracer data. In constructing our equation, we made several minor changes to the biomass requirements previously reported, simply to account for the fact that *i*SR215 does not include certain metabolites and to enable cycling of cofactors. Although the exact requirements of *B. subtilis *for critical metabolites are no doubt different from those of *T. cruzi*, previous work has demonstrated that FBA results are relatively insensitive to changes in biomass component stoichiometric coefficients [72].

### Reaction Deletions

To simulate the effect of deleting reactions, we constrained each of the reactions in turn to have zero flux (a_i _= b_i _= 0) and reran FBA in each case. If no solution was found (biomass reaction flux = 0), the reaction was deemed essential; if a solution was found, the reaction was deemed nonessential.

### Validation

To validate our model, we attempted to replicate findings from the biochemical literature. We used findings from experiments involving targeted disruption of genes or gene products (gene knockouts, RNAi, or drugs to block specific enzymes) in *T. cruzi *or related organisms. Similar manipulations were made using *i*SR215, and the *in silico *and experimental results were compared.

### Epimastigote Specific Metabolism

The life cycle of *T. cruzi *is complex [15]. In the mammalian host, *T. cruzi *replicates intracellularly, as a nearly spherical amastigote, in many cell types. Rupture of the cell releases non-dividing trypomastigotes, which circulate and infect cells in remote organs and tissues. When ingested by blood sucking insect vectors, trypomastigotes transform and replicate as epimastigotes in the insect digestive tract. Epimastigotes are rapidly lysed by complement and are not infective for mammalian hosts. Conditions in the colon and rectum of the reduviid bug induce metacyclogenesis, or differentiation of non-infectious epimastigotes into infectious metacyclic trypomastigotes. The cycle is completed when the bug deposits contaminated excreta near the bite wound and the metacyclic trypomastigotes are mechanically introduced into the host. The metacyclic trypomastigotes are complement resistant and circulate in the host briefly prior to infecting a host cell.

Most biochemical literature relating to *T. cruzi *involves experimental results derived from the insect gut epimastigote stage. We performed both 2D gel analysis and 2D nano LC MS/MS to produce proteomic data for *T. cruzi *epimastigotes. We used these data to further constrain *i*SR215 to be epimastigote-specific. Epimastigotes were grown exponentially in LIT medium, supplemented with 10% fetal calf serum, and different subcellular fractions were collected using subcellular proteome kit (ProteoExtract, Subcellular Proteome Extraction Kit, Calbiochem). Each fraction was submitted to 2D nano LC MS/MS, as previously described [73]. Proteins were identified by searching the MS/MS spectra against our *T. cruzi *database using Bioworks v3.2. Peptide and protein hits were scored and ranked using the new probability-based scoring algorithm incorporated in Bioworks v3.2. Peptides identified as possessing fully tryptic termini with acceptable cross-correlation scores (greater than 1.9 for singly charged peptides, 2.3 for doubly charged peptides, and 3.75 for triply charged peptides), with delta Cn greater than 0.25, and with a probability score of less than 0.0001 were initially accepted. For increased stringency we used reverse database search to adjust the above scores to obtain less than 1% false discovery rate. Proteins that passed this final criterion were accepted for use in the model.

The model resulting from the procedures described in "Metabolic Reconstruction" above was designated as the "full model," since all reactions included could operate at non-zero flux values. We used the proteomics results to apply further constraints to the full model, resulting in a stage-specific "epimastigote model." Specifically, if there was no evidence at the protein level supporting the existence of a specific metabolic reaction in epimastigotes, then that metabolic reaction was constrained to a flux value of zero (a_i _= b_i _= 0, see above). For example, the protein responsible for glucokinase activity (reaction ID "GLUKg") was detected in the epimastigote by our proteomics experiments; therefore, no special constraint was applied to this reaction. On the other hand, the proteins responsible for aldose 1-epimerase activity (A1Eg) were not detected by the proteomics experiments; therefore, this reaction was constrained to have zero flux, since the data suggested no enzymes to catalyze the reaction were expressed. For reactions requiring multiple proteins, we allowed the reaction to occur if there was evidence for the expression of any of the corresponding proteins in the proteomics data.

## Authors' contributions

JP and GB conceived the study, and participated in its design and coordination. SR and JR built the model. SR and AC carried out the simulations and analyzed the data. SR drafted the manuscript. PM, VL, and AL carried out the proteomics experiments.

## Supplementary Material

Additional file 1 **Additional tables**. Four worksheets, including detailed reaction list for *i*SR215, complete listing of metabolite names and abbreviations, full results from proteomics experiments, and lethal reaction deletions (single and non-trivial double) using full model and epimastigote model. Click here for file

Additional file 2 ***i*SR215 in SBML format**. *i*SR215 in SBML format. Click here for file

Additional file 3 ***T. cruzi *core metabolic network**. Map illustrating the core metabolic network in *T. cruzi*. Click here for file

Additional file 4 **Flux distribution for full model**. Graphical depiction of flux distribution for full model. Click here for file

Additional file 5 **Flux distribution for epimastigote model**. Graphical depiction of flux distribution for epimastigote model. Click here for file
