# Supplementary figures and images for: Proteomic and network analysis characterize stage-specific metabolism in Trypanosoma cruzi
Source: BMC Syst Biol. 2009 May 16;3:52. doi: 10.1186/1752-0509-3-52 (PMC2701929; doi:10.1186/1752-0509-3-52)

# Core metabolism of *Trypanosoma cruzi*

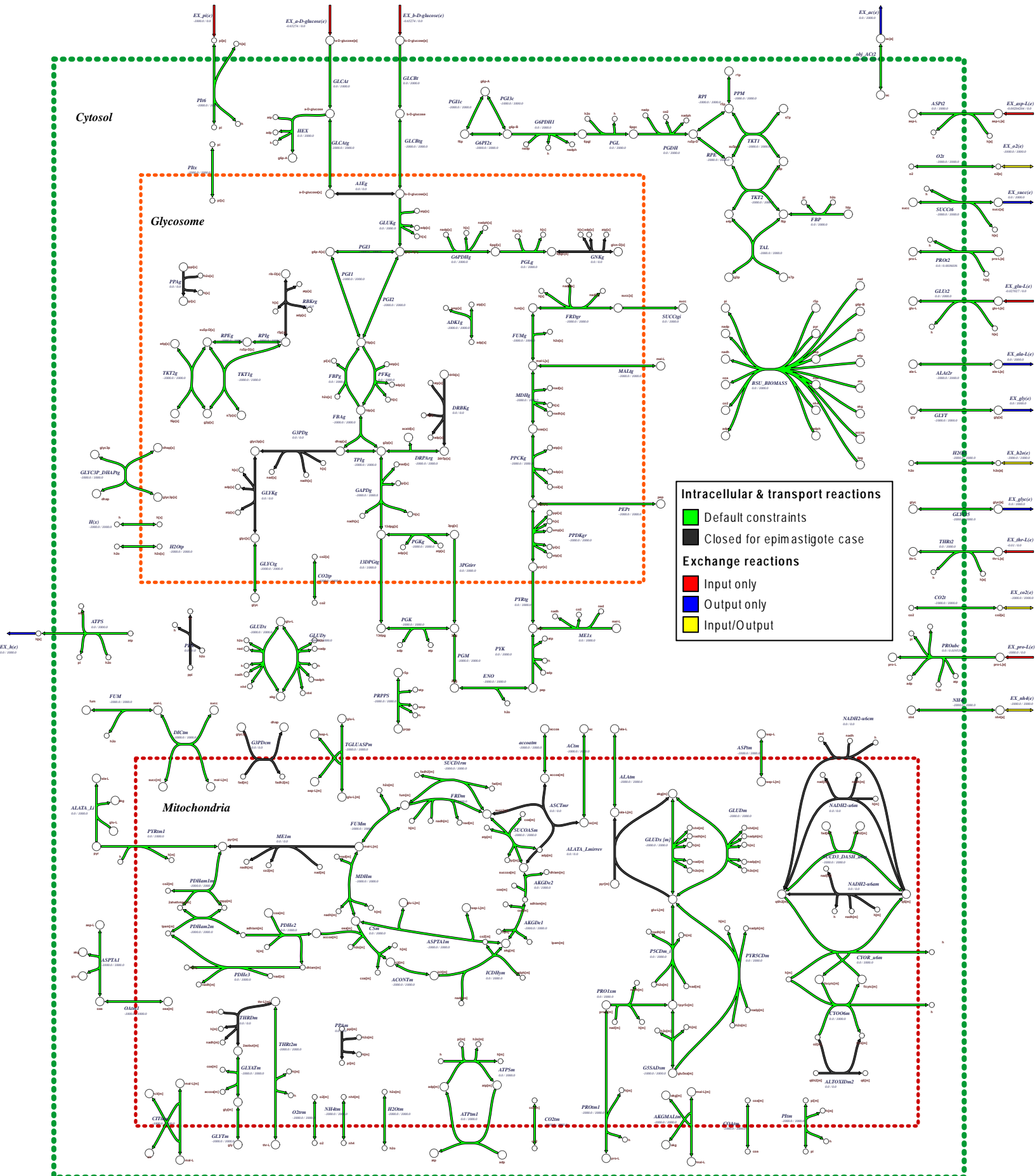

Supplement: Additional file 3 — T. cruzi core metabolic network. Map illustrating the core metabolic network in T. cruzi. [file 1752-0509-3-52-S3.pdf]

# Core metabolism of *Trypanosoma cruzi*

## Flux distribution for "Full" model

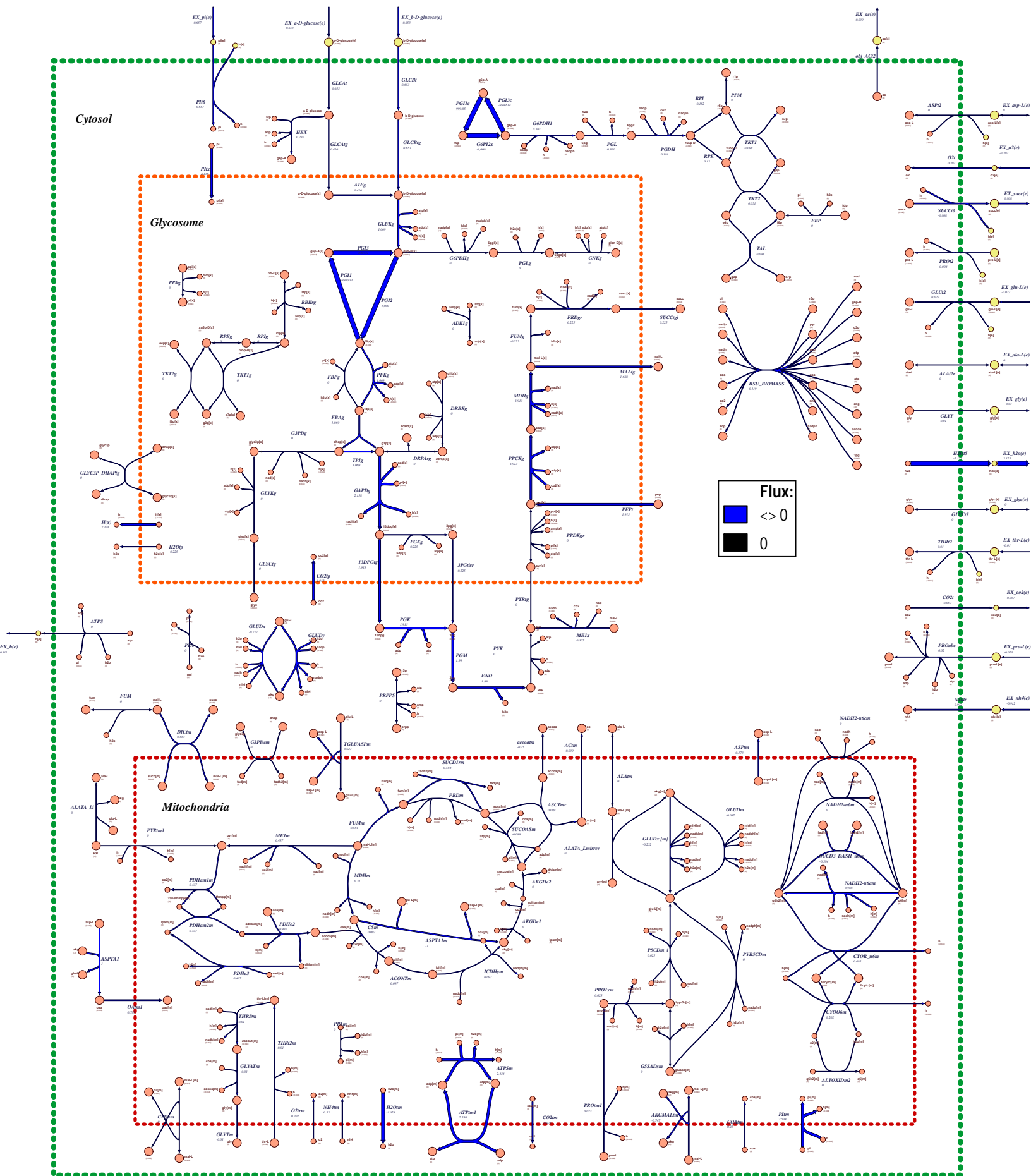

Supplement: Additional file 4 — Flux distribution for full model. Graphical depiction of flux distribution for full model. [file 1752-0509-3-52-S4.pdf]

# Core metabolism of *Trypanosoma cruzi*

## Flux distribution for "Epimastigote" model

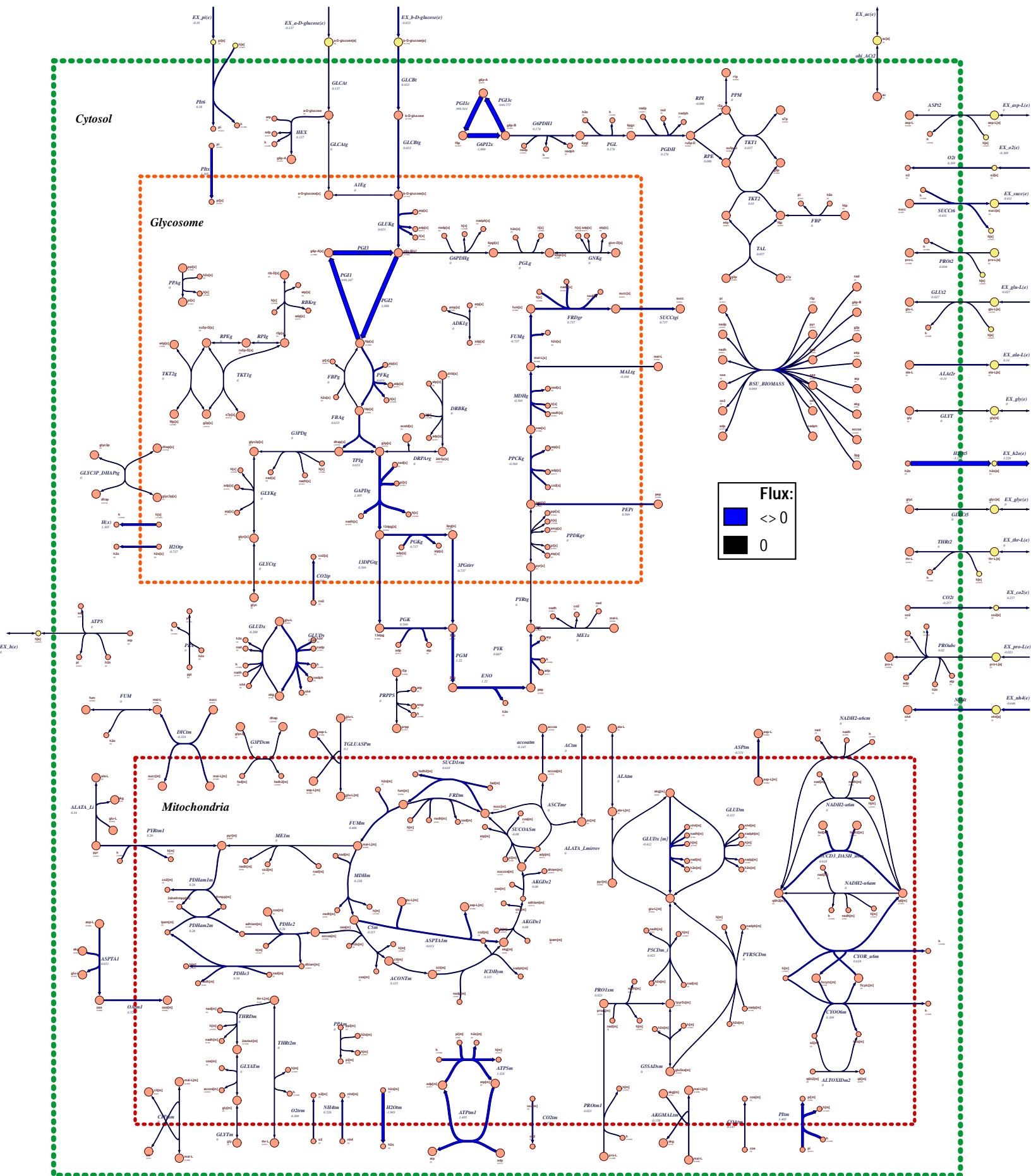

Supplement: Additional file 5 — Flux distribution for epimastigote model. Graphical depiction of flux distribution for epimastigote model. [file 1752-0509-3-52-S5.pdf]
